# Perivascular adipose tissue dysfunction contributes to thoracic aortic aneurysm development

**DOI:** 10.1186/s12933-025-02765-x

**Published:** 2025-05-21

**Authors:** Zhenguo Wang, Wenjuan Mu, Ruiyan Xu, Juan Zhong, Wenhao Xiong, Xiangjie Zhao, Xiubin Liang, Yanhong Guo, Jifeng Zhang, Zhi-Sheng Jiang, Bo Yang, Y. Eugene Chen, Lin Chang

**Affiliations:** 1 Department of Internal Medicine, Cardiovascular Center, University of Michigan Medical Center, Ann Arbor, MI 48109 USA; 2 Institute of Cardiovascular Disease, Key Laboratory for Arteriosclerology of Hunan Province, International Joint Laboratory for Arteriosclerotic Disease Research of Hunan Province, Hengyang Medical School, University of South China, Hengyang, 421001 People’s Republic of China; 3 Key Laboratory of Animal Cellular and Genetics, Engineering of Heilongjiang Province, College of Life Science, Northeast Agricultural University, Harbin, 150030 People’s Republic of China; 4 Department of Cardiac Surgery, Cardiovascular Center, University of Michigan Medical Center, Ann Arbor, MI 48109 USA

**Keywords:** Thoracic aortic aneurysm, Perivascular adipose tissue, PPARg, PRDM16, Decorin

## Abstract

**Background:**

Thoracic aortic aneurysm (TAA) is a life-threatening disease with high morbidity and mortality rates due to fatal complications such as aortic rupture. However, molecular mechanisms underlying TAA pathogenesis remain to be fully elucidated. The aorta is naturally surrounded by perivascular adipose tissue (PVAT), which produces and releases adipokines and other factors in a paracrine manner that are pivotal for vascular physiology and pathophysiology. Under healthy conditions, thoracic PVAT resembles brown adipose tissue (BAT) and maintains vascular homeostasis. In response to pathogenic stimuli, PVAT can undergo whitening and become dysfunctional, contributing to the development of vascular diseases. However, a causal relationship between PVAT dysfunction and TAA pathogenesis, as well as the underlying mechanisms, remain unknown. This study investigated the roles of PPARg (a key determinant of adipogenesis) and PRDM16 (a key determinant of brown adipocyte development) in PVAT on TAA development.

**Methods:**

PVAT samples from TAA patients were collected and evaluated. Mice lacking PVAT and those with dysfunctional PVAT were generated by crossbreeding *Ucp1* promoter-driven Cre mice with *Pparg* floxed mice (brown adipocyte-specific *Pparg* knockout, *Pparg*^BAKO^) and *Prdm16* floxed mice (brown adipocyte-specific *Prdm16* knockout, *Prdm16*^BAKO^), respectively. TAA formation was induced by perivascular application of porcine pancreatic elastase (PPE) and evaluated through histological staining. Luciferase reporter assays and chromatin immunoprecipitation-quantitative PCR (ChIP-qPCR) were used to determine PRDM16 target genes.

**Results:**

We found that PVAT near TAA lesions in patients exhibited reduced expression of browning markers and increased expression of whitening markers. *Pparg*^BAKO^ mice showed impaired PVAT development, while *Prdm16*^BAKO^ mice displayed a loss of browning in PVAT. Both *Pparg*^BAKO^ and *Prdm16*^BAKO^ mice exhibited aggravated TAA formation. We identified decorin, a small proteoglycan of the extracellular matrix, as a transcriptional repressive target gene of PRDM16. The expression of decorin was increased in dysfunctional PVAT and the plasma of TAA patients.

**Conclusions:**

The development and maintenance of brown-like characteristics in PVAT are necessary to protect against TAA formation. PVAT dysfunction contributes to TAA development. Our study provides a promising therapeutic strategy for preventing TAA progression by inducing PVAT browning.

**Supplementary Information:**

The online version contains supplementary material available at 10.1186/s12933-025-02765-x.

## Background

Thoracic aortic aneurysm (TAA) is a life-threatening disease with high morbidity and mortality rates due to fatal complications such as aortic rupture [1]. Current treatments for TAA are limited to open surgical or endovascular repairs [2]. However, these surgical approaches are associated with high perioperative mortality and significant morbidity and poor long-term survival [3]. Pre-clinical studies on pharmacologic agents appeared to show no beneficial outcomes in clinical settings [4], underscoring the urgent need for a deeper understanding of TAA formation and progression to develop alternative and effective drug-based treatments for prevention and early intervention.

Most blood vessels, except for capillaries, cerebral and pulmonary blood vessels, as well as coronary arteries in mice, are surrounded by adipose tissue known as perivascular adipose tissue (PVAT). PVAT exhibits regional heterogeneity: it resembles classical white adipose tissue (WAT) around mesenteric arteries, has brown adipose tissue-like characteristics around the thoracic aorta, and consists of a mixture of both brown and white adipocytes around the abdominal aorta [5]. PVAT produces and releases adipokines and other factors that target the underlying aortic wall in a paracrine manner and are essential for maintaining aortic homeostasis [6, 7]. Normal thoracic PVAT resembles brown adipose tissue (BAT) [5, 8]. PVAT exhibits high plasticity and undergoes dysfunctional changes in response to various stimuli and pathological conditions, including cardiovascular factors like angiotensin II (Ang II) and metabolic disorders such as obesity and diabetes [5, 9, 10]. Recent studies indicate that PVAT dysfunction is highly associated with abdominal aortic aneurysm (AAA) pathogenesis in both humans and experimental animals [11–15]. Additionally, modulation of PVAT function by mirabegron, a b3-adrenergic receptor agonist, has been shown to prevent aortic dissection/aneurysm [16]. However, a causal relationship between PVAT and TAA and the possible underlying mechanisms remain to be elucidated.

Peroxisome proliferator-activated receptor gamma (PPARg) is the master regulator of adipogenesis and is both necessary and sufficient for adipocyte differentiation [17]. Previously, we reported that PPARg ablation in brown adipocytes or vascular smooth muscle cells (VSMCs) significantly impaired PVAT function, leading to enhanced atherosclerosis in mice [8, 18]. PPARg directly interacts with PR-domain containing 16 (PRDM16), which is a determinant of the brown gene program in adipocytes [19, 20]. PRDM16 deficiency in brown adipocytes results in whitening of BAT and impaired BAT development and function in mice [21, 22]. However, the effects of PPARg and PRDM16 in PVAT adipocytes on aortic aneurysm remain unknown. In this study, we investigated how dysfunction in PVAT, caused by deficiencies in either PPARg or PRDM16, contributes to TAA pathogenesis.

## Methods

### Human samples

Human aorta and PVAT specimens were obtained from both the aneurysmal and adjacent normal regions in TAA patients undergoing scheduled open surgical repair at the University of Michigan Cardiovascular Center (Patients’ information is provided in Supplemental Table 1). For additional information, see “Ethical approval.” For histological analysis, the aortic and PVAT samples were fixed in 10% buffered formalin in PBS, followed by embedding in paraffin, sectioning and staining by the Pathology Core at the University of Michigan. Tissue sections (5 μm thick) were deparaffinized, rehydrated, and subjected to Verhoeff-Van Gieson (VVG) staining (catalog #ab150667, Abcam) or hematoxylin and eosin (H&E) staining [23].

### Animals

Brown adipocyte-specific *Pparg* knockout (*Pparg*^BAKO^) or *Prdm16* knockout (*Prdm16*^BAKO^) mice were generated by crossbreeding *Ucp1* promoter-driven Cre mice (strain number 024670, The Jackson Laboratory) with *Pparg* floxed mice (strain number 004584, The Jackson Laboratory) and *Prdm16* floxed mice (strain number 024992, The Jackson Laboratory), respectively. All mice were maintained on a C57BL/6J background and housed under regular housing conditions (12-hour light/dark cycle, 20–23 °C) with free access to regular chow diet and tap water.

### TAA induction

The procedure for TAA induction was similar to those described previously [24]. In brief, twelve-week-old male mice were anesthetized with isoflurane inhalation. A midline incision on the sternum was made, and the thymus was pushed aside to expose the aortic arch without opening the pleura (Supplemental Fig. 1A). The advantage of this procedure is that a ventilator is not required. The PVAT surrounding the aortic arch was then bluntly disassociated. The aortic arch was then covered with a piece of gauze (1 × 1 mm) soaked in porcine pancreatic elastase (PPE, catalog #E1250, Sigma-Aldrich) for 10 min. Then the gauze was removed, and the aortic arch was washed with 37 °C-warmed normal saline 3 times. The PVAT was sutured with 9–0 monofilament (REF AK-0109, Surgical Specialties Corporation) to secure it to its original location. The muscle wound was sutured by 6– 0 suture, and the skin wound was closed by clips. After 2 weeks, mice were euthanized, and the TAA lesion was evaluated (Supplemental Fig. 1B–C). Photos of TAA lesions were captured and analyzed using ImageJ. Cross-sections of paraffin-embedded aortic arch were prepared, and Masson’s trichrome staining and H&E staining were performed by the Pathology Core Facility (RRID: SCR_018823) at the University of Michigan. VVG staining was performed using the Elastic Stain Kit (catalog #ab150667, Abcam). Images were captured with a microscope (BZ-X800, KEYENCE). Quantification of the degree of elastic fiber degradation levels in the aortic wall was shown in Supplemental Fig. 2.

### Echocardiography in mice

The Physiology Phenotyping Core at the University of Michigan performed the echocardiography procedure. In brief, mice were anesthetized with 2% isoflurane inhalation. Chest hair was removed using a depilatory cream, and ultrasound gel was applied. The high-frequency transducer was then positioned on the chest to obtain standard aortic views for aortic diameter measurements.

### Whole-body metabolism measurement by indirect calorimetry

Michigan Mouse Metabolic Phenotyping Center performed the whole-body metabolism. In brief, the mice were acclimated to single housing in an indirect calorimetry system (Comprehensive Lab Animal Monitoring System, Columbus) under controlled environmental conditions: 22 °C ambient temperature, 39% humidity, and a 12-hour light/dark cycle (lights on at 6:00 AM). The airflow rate through the chambers was adjusted to maintain an O_2_ differential of approximately 0.3% under resting conditions. Oxygen consumption (VO_2_) and carbon dioxide production (VCO_2_) were sampled for 5 s at 20-min intervals. The respiratory exchange ratio (RER) and total energy expenditure (EE) were calculated based on VO_2_ and VCO_2_ measurements.

### Insulin tolerance test (ITT) and glucose tolerance test (GTT)

For the ITT, the mice were fasted for 4 h before receiving an intraperitoneal injection of insulin (Humulin R) at a dose of 0.5 U/kg body weight. For the GTT, mice were fasted for 6 h before oral gavage administration of glucose (2 g/kg body weight) in drinking water. Blood samples were collected via tail vein puncture at baseline and every 30 min for up to 2 h following insulin or glucose administration. Blood glucose levels were measured using a blood glucose meter and test strips (Contour Next EZ).

### Quantitative polymerase chain reaction (qPCR)

qPCR was performed as previously described [24]. Briefly, total RNA was extracted using the RNeasy Mini kit (catalog #74106, Qiagen) and 2 µg of total RNA was subjected to cDNA synthesis with the SuperScript III First-Strand Synthesis System (catalog #18080, Life Technologies). Relative mRNA expression was calculated by normalization to 18 S rRNA levels. The number of biological replicates is specified in the figure legends. All qPCR analyses were performed in at least two independent experimental runs, and representative results are presented. Primer sequences used in this study are listed in Supplemental Table 2.

### Western blot

Western blot was performed as previously described [25]. Briefly, protein lysates from adipose tissue or cultured cells were prepared with RIPA buffer (catalog #89901, Thermo Fisher Scientific) supplemented with protease inhibitor cocktail (catalog #11873580001, Roche). Protein concentrations were determined using the Pierce™ BCA protein assay kits (catalog #23227, Thermo Scientific). Equal amounts of protein lysates (10 µg) were resolved by SDS-PAGE and transferred to nitrocellulose membranes (catalog #1620115, Bio-Rad). The membranes were blocked in 5% non-fat dry milk dissolved in Tris-buffered saline containing 0.1% Tween 20 for 1 h at room temperature and were probed with primary antibodies overnight at 4 °C: anti-Tubulin (1:1000; catalog #2148, Cell Signaling Technology), anti-PRDM16 (1:1000; catalog #ab106410 or #ab303534, Abcam), anti-b-actin (1:2000; catalog #4967, Abcam). The signals were captured and quantified using Image Studio (version 3.1, Odyssey CLx).

#### Evaluation of apoptosis

Mouse primary VSMCs were isolated from the thoracic aorta of C57BL/6J mice and cultured as previously described [26]. Conditioned medium of PVAT from *Prdm16*^BAKO^ and littermate control mice was collected by incubating the PVAT in DMEM at 37 °C for 24 h in an incubator with 7% CO_2_. Next, mouse primary VSMCs were treated with PVAT conditioned medium for 24 and 48 h. Western blot analysis was used to determine the cleavage of PARP using the anti-PARP antibody (catalog #9542, Cell Signaling Technology). For the detection of cell apoptosis in paraffin-embedded tissue samples, a TUNEL Assay Kit (catalog #ab206386, Abcam) was used following the manufacturer’s instruction. Briefly, tissue 5-µm-thick sections were deparaffinized and treated with proteinase K, followed by hydrogen peroxide. The sections were then incubated with terminal deoxynucleotidyl transferase (TdT) and anti-digoxigenin-peroxidase, developed with diaminobenzidine (DAB), and counterstained with hematoxylin.

#### Immunohistochemistry for macrophage

The sections were deparaffinized, rehydrated, and boiled for 15 min in Citrate Buffer (pH 6.0, catalog #00-5000, Invitrogen) for epitope retrieval. Then the sections were blocked with 5% donkey serum in PBS for 1 h and incubated with primary antibodies against F4/80 (catalog #ab16911, abcam), or species-matched normal IgG at 4 °C overnight. After washing with PBS, the sections were processed using an IHC Kit (catalog #ab64261, abcam) before image collection with an Olympus DP73 microscope.

#### Measurement of decorin levels by ELISA

Conditioned medium of PVAT from *Prdm16*^BAKO^ and littermate control mice was collected by incubating the PVAT in DMEM at 37 °C for 24 h in an incubator with 7% CO_2_. Decorin levels in the conditioned medium were determined using an ELISA kit (catalog #EMDCN, Invitrogen, ThermoFisher). Decorin levels in human plasma were determined using an ELISA kit (catalog #EHDCN, Invitrogen, ThermoFisher).

#### Luciferase reporter assay

The promoter region of decorin was amplified by PCR and cloned into the pGL4.11[luc2P] vector (catalog #E6661, Promega). Amplicons were validated by Sanger sequencing. The luciferase plasmids along with PPARg or PRDM16 expression vector (pcDNA3.1, Invitrogen) were then co-transfected into HEK293T cells using Lipofectamine 2000 (catalog #11668019, ThermoFisher Scientific) following the manufacturer’s instructions. 24 h later, cells were harvested in passive lysis buffer and luciferase activities were determined by dual luciferase assay (catalog #E1910, Promega). Data are presented as relative luciferase activity against Renilla activity.

#### Chromatin Immunoprecipitation (ChIP) assay

ChIP assay was performed with the SimpleChIP^®^ Enzymatic Chromatin IP Kit (catalog #9003, Cell Signaling Technology), according to the manufacturer’s instructions. Briefly, preadipocytes in mouse PVAT were isolated as previously described [27], and were infected with 10 MOI lentivirus carrying empty vector or PRDM16 for 48 h. The cells were then treated with 2 µg/mL puromycin (catalog #P9620, Sigma-Aldrich) for one week. The resulting stably expressed cells were incubated with 1% paraformaldehyde (catalog #158127, Sigma-Aldrich) for 10 min at room temperature to crosslink proteins with DNA, followed by neutralization with glycine for 5 min. The nuclei were prepared and digested with Micrococcal Nuclease for 20 min at 37 °C, followed by sonication (Branson Sonifier SLPe, 20 s of 35% amplification, 3 times). The purified sheared chromatin was then immunoprecipitated with anti-FLAG antibody (1 µg; catalog #14793, Cell Signaling Technology) or equal amount of normal IgG (catalog #2729, Cell Signaling Technology) overnight at 4 °C with gentle rotation. The precipitated DNA was extensively washed with low-salt buffer and high-salt buffer. The eluted protein-DNA complexes were reversed via incubation with proteinase K for 2 h at 65 °C. Purified DNA was used for qPCR analysis. ChIP-qPCR data are expressed as percentage input, which is calculated by the following equation: (percent input = 2% x 2(Ct(2% input sample)-Ct(IP sample)). Primer sequences used in this study are listed in Supplemental Table 2.

### Statistics

Statistical analyses were performed using GraphPad Prism software (version 10.3.1). All data were assessed for variance and normality. The sample size was determined based on preliminary studies and our previous publications [24, 28]. The 2-tailed Student’s t test was used for comparisons between two groups. One-way ANOVA or two-way ANOVA followed by Holm-Šidák multiple-comparison test was used for comparisons among three or more groups, as specified in the figure legends. A *p* value less than 0.05 was considered significant.

## Results

### Loss of Browning of PVAT near TAA lesions in patients

**Fig. 1:**
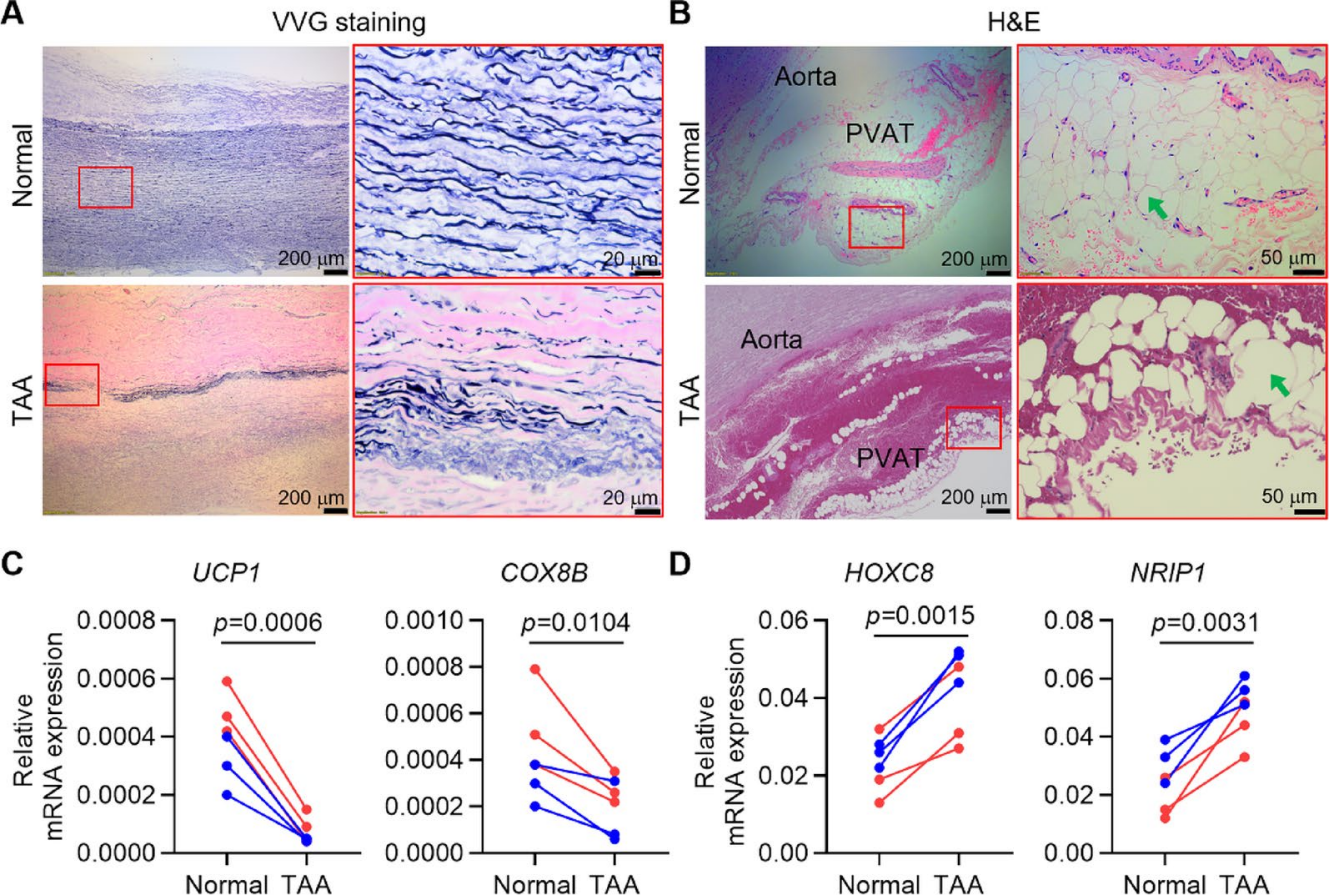
Loss of browning of PVAT near TAA lesions in patients. **A** Verhoeff-Van Gieson (VVG) staining reveals severe elastin loss in the thoracic aorta at the TAA lesion region in patients. **B** H&E staining shows the structure of PVAT adjacent to the thoracic aorta. Green arrows indicate adipocytes. **C–D** The mRNA expression of marker genes of browning **C** or whitening **D** in PVAT samples were determined by qPCR analysis. *UCP1*, uncoupling protein 1; *COX8B*, cytochrome c oxidase subunit 8B, mitochondrial; *HOXC8*, homeobox protein Hox-C8; *NRIP1*, nuclear receptor-interacting protein 1. Blue and red dots represent male and female patients, respectively. *n* = 6 patients. Data are presented as mean ± SEM. *p* values were calculated using 2-tailed paired Student’s *t* test

To elucidate the role of PVAT in TAA pathogenesis, we collected both aortic and PVAT samples from the aneurysmal lesion regions and relatively normal regions of TAA patients undergoing surgical repair (Supplemental Table 1). Verhoeff-Van Gieson (VVG) staining of the thoracic aorta showed severe loss of elastic fibers in the aneurysmal regions compared to relative normal regions (Fig. 1A). H&E staining of PVAT showed larger adipocytes in PVAT near TAA lesions compared to relatively normal areas (Fig. 1B). QPCR analysis revealed decreased mRNA expression of browning marker genes (such as *UCP1* and *COX8B*) and increased mRNA expression of whitening marker genes (such as *HOXC8* and *NRIP1*) in PVAT near TAA lesions (Fig. 1C–D). Therefore, PVAT near TAA lesions appeared to be dysfunctional, characterized by the loss of browning features.

### Enhanced elastase-induced TAA formation in mice lacking normal PVAT

**Fig. 2:**
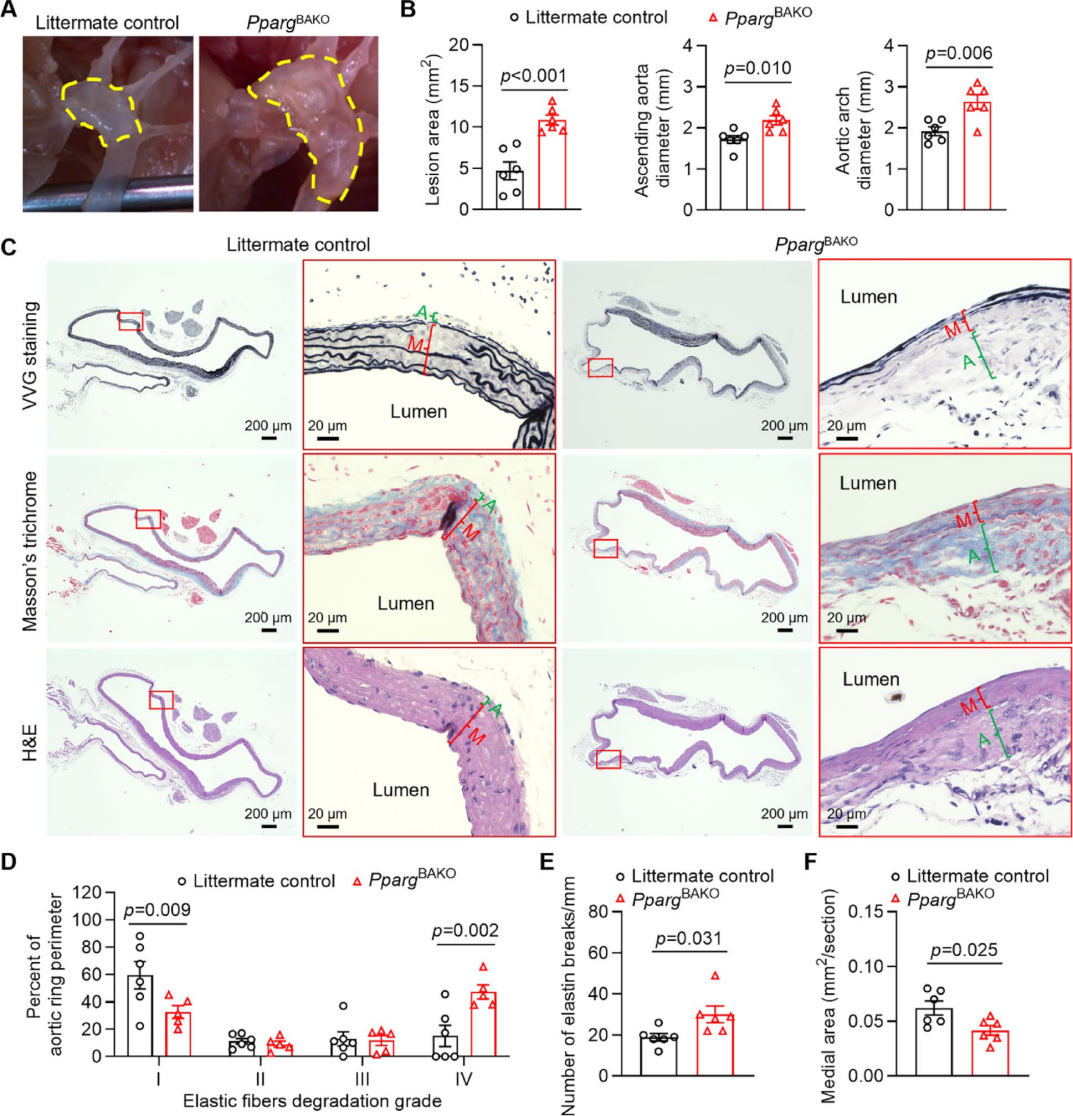
Enhanced TAA formation in *Pparg*^BAKO^ mice lacking normal PVAT. Twelve-week-old male brown adipocyte-specific *Pparg* knockout (*Pparg*^BAKO^) mice and littermate control mice underwent TAA induction via periadventitial application of porcine pancreatic elastase (PPE). Two weeks later, TAA formation was evaluated. **A** Representative overview of TAA lesion regions, indicated by yellow dashed lines. **B** Measurements of the lesion area and diameters of the ascending aorta and trans-aortic arch. *n* = 6 per group. **C** Representative Verhoeff–Van Gieson (VVG) staining, Masson’s trichrome staining, and H&E staining results of the aortic arch region. The adventitia (green A) and media (red M) layers of the aorta were marked. **D** Quantification of the degree of elastic fiber degradation levels in the aortic arch region. *n* = 6 per group. **E** The number of elastin breaks in the Grade I-III regions was quantified by counting the breaks observed in the cross-section. To account for variations in TAA lesion size and area, the total number of elastin breaks was normalized by dividing by the length of Grade I-III along the perimeter of the cross-section. *n* = 6 per group. **F** Measurements of the medial layer area. *n* = 6 per group. Data are presented as mean ± SEM. *p* values were calculated using 2-tailed Student’s *t* test **B**, **E** and **F** or two-way ANOVA with Holm-Šidák multiple-comparison test (**D**)

We next generated brown adipocyte-specific *Pparg* knockout (*Pparg*^BAKO^) mice by crossbreeding *Pparg* floxed mice with *Ucp1* promoter-driven Cre mice, which have been shown lacking normal PVAT [18]. Under basal conditions, the aortic diameter of the ascending aorta, aortic arch, and descending aorta, measured by echocardiographic analysis, were comparable between *Pparg*^BAKO^ mice and littermate control mice (Supplemental Fig. 3). Next, *Pparg*^BAKO^ mice and littermate control mice were subjected to TAA induction via perivascular application of PPE. Our results demonstrated that *Pparg*^BAKO^ mice developed more severe TAA lesion compared to littermate control mice, as evidenced by significantly increased lesion areas, ascending aortic and trans-aortic diameters (Fig. 2A–B). Furthermore, *Pparg*^BAKO^ mice exhibited greater fragmentation and degradation of elastin fibers, along with increased collagen deposition in the adventitia layer and thinner media layer (Fig. 2C). Quantitative assessment of the degree of elastic fiber degradation revealed that *Pparg*^BAKO^ mice had significantly lower percentages of well-organized elastic fibers and markedly higher percentages of severely degraded elastic fibers (Fig. 2D-E). The area of the medial layer was also significantly decreased in *Pparg*^BAKO^ mice compared to littermate control mice (Fig. 2F). Because the PPE was not applied to the ascending and descending aorta, the elastic fibers in both regions remained largely intact (Supplemental Fig. 4). These findings indicate that the absence of normal PVAT exacerbates TAA formation in mice.

### PRDM16 is associated with PVAT function

**Fig. 3:**
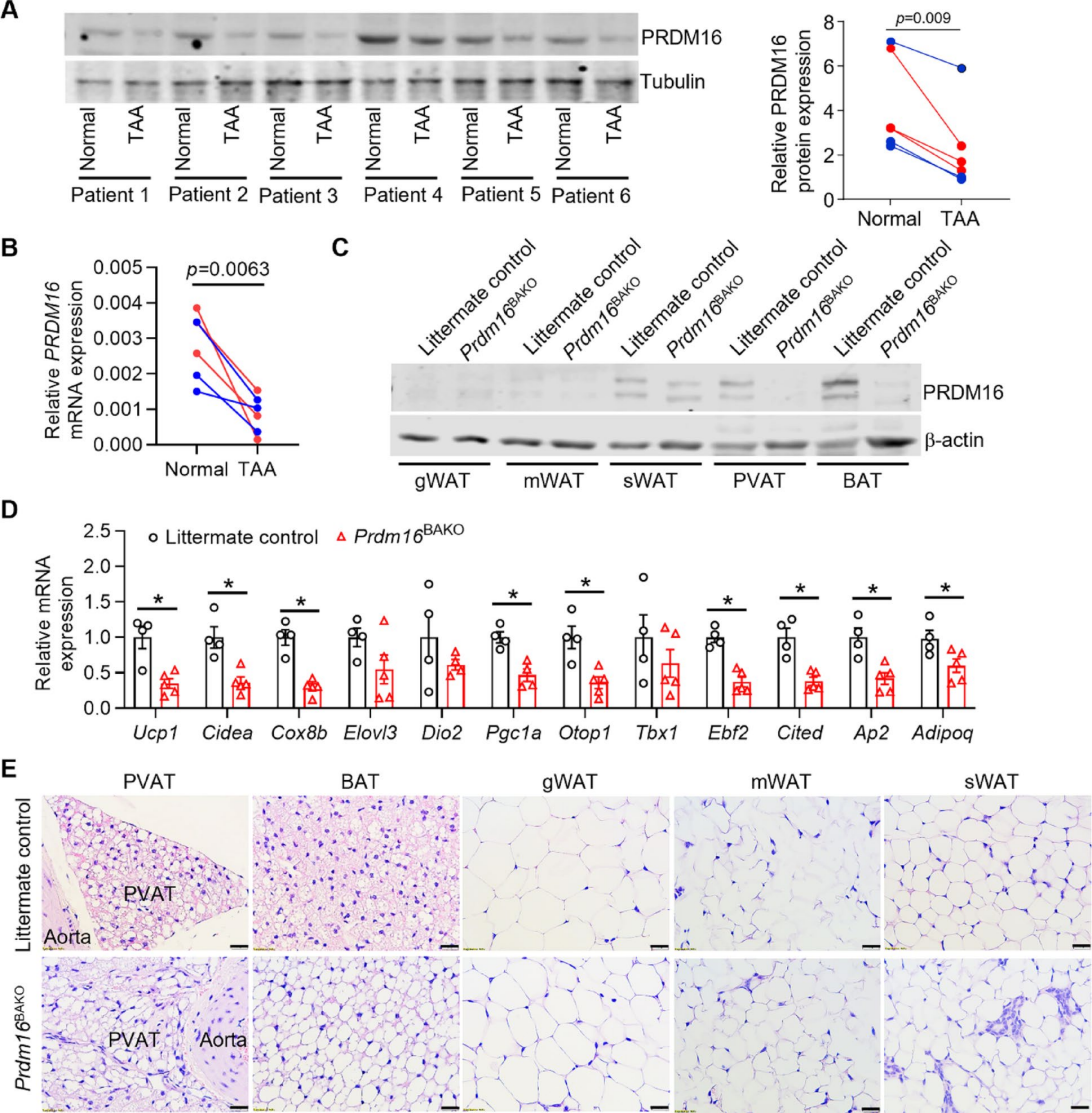
PRDM16 is associated with PVAT functional maintenance. **A** PRDM16 protein expression in PVAT samples from TAA lesions compared to normal regions in TAA patients were determined by western blot (left panel, anti-PRDM16, Abcam catalog #ab106410; anti-Tubulin, Cell Signaling Technology catalog #2148). Quantification results are shown in the right panel. TAA lesions were located in the ascending aorta (for patients 2, 4, and 5) and descending aorta (for patients 1, 3, and 6). *n* = 6 per group. **B** PRDM16 mRNA expression in the same PVAT samples as in **A** were determined by qPCR analysis. *n* = 6 per group. **C** Western blot analysis of PRDM16 protein expression in gonadal WAT (gWAT), mesenteric (mWAT), subcutaneous WAT (sWAT), thoracic PVAT, and BAT from brown adipocyte-specific *Prdm16* knockout (*Prdm16*^BAKO^) mice and littermate control mice (anti-PRDM16, Abcam catalog #ab303534; anti-b-actin, Abcam catalog #4967). **D** The mRNA expression of brown adipocyte-selective genes was determined by qPCR. *n* = 4–5 per group. **E** H&E staining of sections from PVAT, BAT, gWAT, mWAT, and sWAT. Scale bars: 20 μm. Data are presented as mean ± SEM. *p* values were calculated using 2-tailed paired Student’s *t* test **A–B** or 2-tailed Student’s *t* test (**D**); *p < 0.05

It is well-established that PRDM16 is a dominant factor in the fate determination of brown adipocytes [19, 20]. To investigate whether PRDM16 participates in PVAT functional maintenance in TAA patients, we determined the protein levels and mRNA expression of PRDM16 in PVAT near TAA lesions and non-TAA regions. We found that both the protein levels and mRNA expression of PRDM16 were significantly reduced in PVAT near TAA lesions compared to PVAT of non-TAA regions (Fig. 3A–B). To further explore this, we generated brown adipocyte-specific *Prdm16* knockout (*Prdm16*^BAKO^) mice by crossbreeding *Prdm16* floxed mice with *Ucp1* promoter-driven Cre mice. The knockout efficiency of PRDM16 in brown-like adipose tissue, including thoracic PVAT and BAT, was confirmed by western blot analysis (Fig. 3C). The expression of brown adipocyte-selective genes, including *Ucp1*, *Cidea*, *Cox8b*, *Pgc1a*, *Otop1*, *Ebf2*, and *Cited1*, as well as genes for AP2 (adipocyte differentiation marker) and adiponectin (an adipokine), was significantly decreased in the thoracic PVAT from *Prdm16*^BAKO^ mice compared to littermate control mice (Fig. 3D). Deficiency of PRDM16 in brown adipocytes also significantly increased macrophage infiltration and expressions of inflammatory genes in PVAT (Supplemental Fig. 5). Additionally, we observed that lipid droplets in the thoracic PVAT and BAT from *Prdm16*^BAKO^ mice were significantly larger than those in littermate control mice (Fig. 3E). However, lipid droplet size in white adipose tissue (WAT), including gonadal WAT (gWAT), mesenteric WAT (mWAT), and subcutaneous WAT (sWAT), was comparable between the two groups (Fig. 3E). Moreover, *Prdm16*^BAKO^ mice exhibited no significant differences in oxygen consumption, carbon dioxide production, respiratory exchange ratio, total energy expenditure, insulin sensitivity, and glucose disposal compared to littermate control mice (Supplemental Fig. 6), indicating that PRDM16 deficiency in brown adipocytes does not impact whole-body metabolism. These findings suggest that loss of PRDM16 function in PVAT adipocytes induces PVAT whitening, which may contribute to the progression of TAA.

### PRDM16 deficiency in PVAT exacerbates TAA formation in mice

**Fig. 4:**
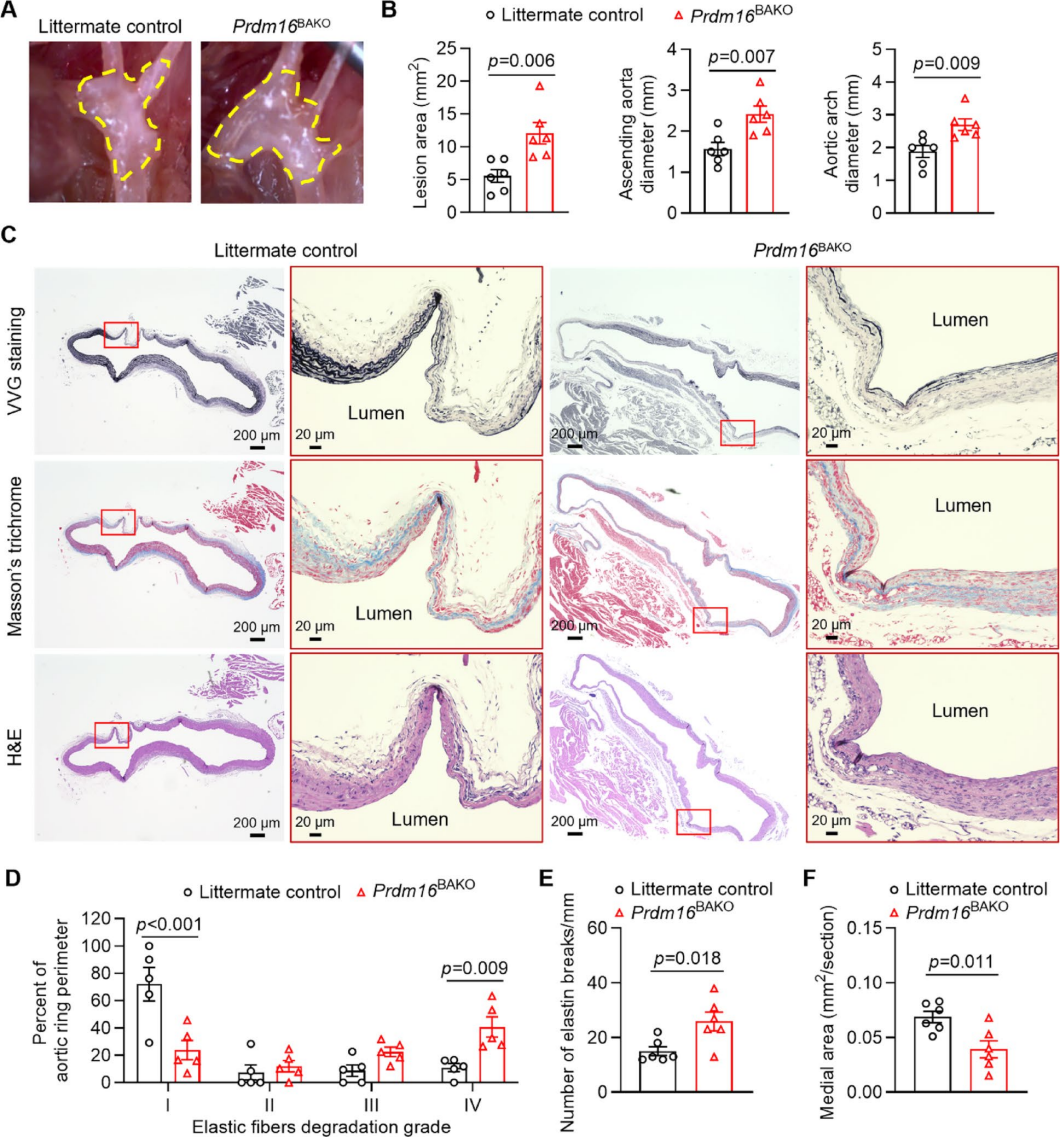
Exacerbated TAA formation in *Prdm16*^BAKO^ mice. Twelve-week-old male brown adipocyte-specific *Prdm16* knockout (*Prdm16*^BAKO^) mice and littermate control mice underwent TAA induction via periadventitial application of porcine pancreatic elastase (PPE). Two weeks later, TAA formation was evaluated. **A** Representative images of TAA lesions, indicated by yellow dashed lines. **B** Measurements of the lesion area and diameters of the ascending aorta and trans-aortic arch. *n* = 6 per group. **C** Representative Verhoeff-Van Gieson (VVG) staining, Masson’s trichrome staining, and H&E staining results of the aortic arch region. **D** Quantification of the degree of elastic fiber degradation levels in the aortic arch region. *n* = 6 per group. **E** The number of elastin breaks in the Grade I-III regions was quantified by counting the breaks observed in the cross-section. To account for variations in TAA lesion size and area, the total number of elastin breaks was normalized by dividing by the length of Grade I-III along the perimeter of the cross-section. *n* = 6 per group. **F** Measurements of the medial layer area. *n* = 6 per group. Data are presented as mean ± SEM. *p* values were calculated using 2-tailed Student’s *t* test (**B**, **E** and **F**) or two-way ANOVA with Holm-Šidák multiple-comparison test (**D**)

We next determined whether PVAT dysfunction contributes to aggregated TAA formation. Under basal conditions, the aortic diameter of the ascending aorta, aortic arch, and descending aorta, measured by echocardiographic analysis, were comparable between *Prdm16*^BAKO^ mice and littermate control mice (Supplemental Fig. 7). *Prdm16*^BAKO^ mice and littermate control mice were then subjected to TAA induction by perivascular application of PPE. Our results demonstrated that *Prdm16*^BAKO^ mice exhibited aggregated TAA formation, with significantly increased TAA lesion area, ascending aortic and maximal trans-aortic diameter (Fig. 4A–B). Moreover, *Prdm16*^BAKO^ mice showed increased fragmentation and degradation of elastin fibers (Fig. 4C). Quantitative assessment of the degree of elastic fiber degradation revealed that *Prdm16*^BAKO^ mice showed significantly decreased percentages of well-organized elastic fibers, but markedly increased percentages of greatly degraded elastic fibers (Fig. 4D-E). Additionally, the area of the medial layer was significantly decreased in *Prdm16*^BAKO^ mice compared to littermate control mice (Fig. 4F). In contrast, the elastic fibers in the adjacent ascending and descending aorta remained largely intact, with a few occasional fiber breakages (Supplemental Fig. 8). These data indicate that PRDM16 deficiency-induced PVAT dysfunction exacerbates TAA formation.

### Thoracic PVAT regulates VSMCs apoptosis

Apoptosis of VSMCs is a crucial feature of TAA [29]. We observed a higher level of cell apoptosis in both the PVAT and the underlying aorta of *Prdm16*^BAKO^ compared to littermate controls, as determined by the TUNEL assay (Supplemental Fig. 9A). Previous studies have indicated that co-culture of brown-like PVAT with VSMCs prevented VSMCs apoptosis [15]. To verify whether loss of PRDM16 function in PVAT induces VSMCs apoptosis, we cultured primary mouse VSMCs in conditioned medium from the PVAT of *Prdm16*^BAKO^ mice and littermate control mice and evaluated apoptosis of mouse VSMCs. We observed that PRDM16 deficiency in PVAT resulted in significantly increased cleaved PARP (apoptosis marker) in VSMCs (Supplemental Fig. 9B). These results indicate that PVAT dysfunction induces VSMCs apoptosis, potentially contributing to TAA pathogenesis.

#### PRDM16 transcriptionally represses the expression of decorin

**Fig. 5:**
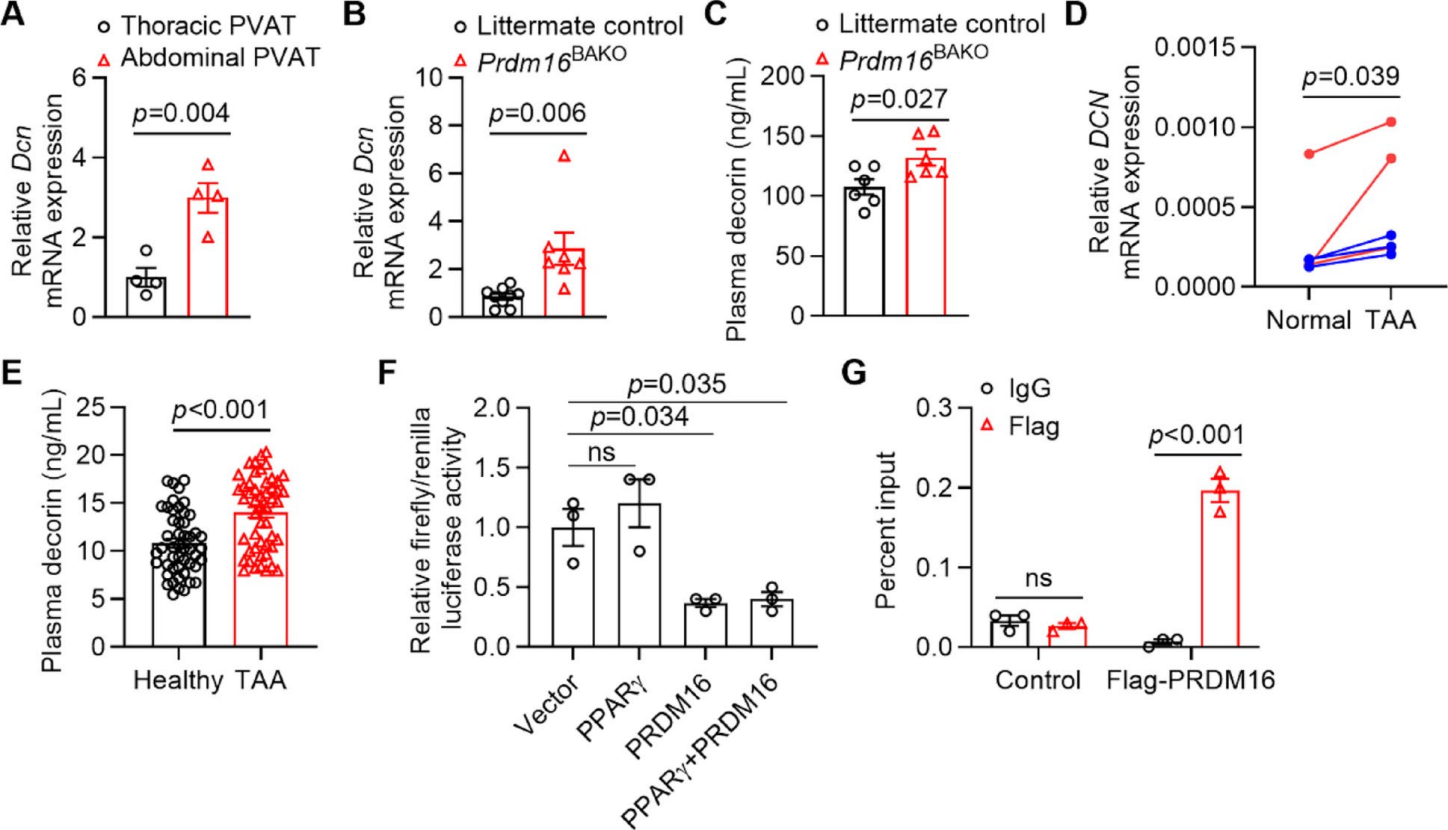
PRDM16 transcriptionally represses the expression of decorin. **A** Decorin mRNA expression in the thoracic PVAT and abdominal PVAT from male C57BL/6J mice. *n* = 4 per group. **B** Decorin mRNA expression in thoracic PVAT samples from *Prdm16*^BAKO^ mice and littermate control mice was determined by qPCR analysis. *n* = 7–9 per group. **C** The plasma decorin levels in *Prdm16*^BAKO^ mice and littermate controls were determined by ELISA. *n* = 6 per group. **D** Decorin mRNA expression in PVAT from TAA lesions and normal regions in TAA patients was determined by qPCR analysis. *n* = 6 per group. **E** Plasma decorin levels in TAA patients compared with healthy individuals were determined by ELISA. *n* = 49–50 per group. **F** Luciferase assay in HEK293T cells transfected with *Dcn* promoter-driven luciferase reporters and PRDM16 or PPARg expression plasmids. *n* = 3 per group. **G** Preadipocytes isolated from thoracic PVAT were infected with lentivirus carrying either empty vector (control) or PRDM16 (Flag-PRDM16). ChIP assay followed by qPCR analysis was performed to determine the binding of PRDM16 to the promoter region of *Dcn*. *n* = 3 per group. Data are presented as mean ± SEM. *p* values were calculated using 2-tailed Student’s *t* test (**A**–**C**), 2-tailed paired Student’s *t* test (**D**), Mann-Whitney U test (**E**), one-way ANOVA with Holm-Šidák multiple-comparison test (**F**), or two-way ANOVA with Holm-Šidák multiple-comparison test (**G**)

PVAT uniquely crosstalk with VSMCs *via* paracrine signaling [30]. To identify the paracrine factor(s) responsible for the pro-apoptotic features of dysfunctional PVAT, we systematically analyzed the expression of adipokines and other factors in PVAT. We found that decorin exhibited higher mRNA expression in white-like abdominal PVAT than brown-like thoracic PVAT (Fig. 5A), suggesting a negative association with browning. Decorin is a small proteoglycan of extracellular matrix (ECM), which has been implicated in the development of AAA [31, 32]. Therefore, we determined the expression of decorin in dysfunctional PVAT from humans and mice. Our results revealed that the mRNA expression of decorin in thoracic PVAT and the plasma decorin levels were significantly increased in *Prdm16*^BAKO^ mice compared to control mice (Fig. 5B–C). Similar results were observed in humans, with the mRNA expression of decorin significantly increased in PVAT near TAA lesions compared to non-TAA regions (Fig. 5D). Furthermore, the plasma decorin levels were markedly elevated in TAA patients compared with healthy individuals (Fig. 5E).

PRDM16 is a transcription factor enriched in brown adipocytes [19]. To determine whether PRDM16 directly regulates decorin expression, we performed luciferase assay and ChIP-qPCR analysis. We found that PRDM16 expression significantly inhibited the luciferase activities driven by decorin promoter, independent of PPARg co-expression (Fig. 5F). In addition, ChIP-qPCR analysis revealed that PRDM16 binding signals were markedly enriched in the promoter region of decorin (Fig. 5G), suggesting a direct transcriptional regulation of decorin by PRDM16. In summary, these data indicate that decorin is a transcriptional repressive target gene of PRDM16 in PVAT adipocytes.

## Discussion

Since the rediscovery of functional BAT in adult humans, significant scientific efforts have been dedicated to identifying molecular mechanisms that promote the phenotypic transformation of white adipocytes into brown-like (beige) adipocytes, a process known as “browning” [33]. Similar to classical brown adipocytes, beige adipocytes enhance energy expenditure due to high levels of UCP1 in the mitochondria [34]. Consequently, strategies aimed at inducing WAT browning may contribute to the prevention of obesity and CVD by increasing thermogenesis and energy expenditure [35]. PVAT is an integral, functional layer of blood vessels responsible for maintaining vascular homeostasis. While PVAT predominantly consists of WAT in resistance blood vessels, it comprises brown or beige adipose tissue in large blood vessels [36]. The anatomical structure of PVAT within the vessel wall, along with the intrinsic energy expenditure capabilities of brown or beige adipocytes, underscores the significant physiological roles of PVAT and its potential as a therapeutic target for CVD [37]. However, upon whitening of beige adipocyte, its thermogenic function is diminished, leading to macrophage infiltration and adipocyte dysfunction as a result of increased secretion of inflammatory factors [38]. This alteration in the balance of PVAT-released factors can detrimentally impact vascular function by promoting dysfunction of VSMCs and endothelial cells, potentially leading to CVD [9, 30]. Several large-scale epidemiological studies have demonstrated a positive association between PVAT volume and the prevalence of CVD and coronary artery disease (CAD) [39, 40]. A recent systematic review and meta-analysis involving a total of 7797 patients further established that higher values of the PVAT fat attenuation index (FAI), which is the average reduction in the signal of adipose tissue within a volume of interest as measured from reconstructed computerized tomography scanning, offered incremental prognostic value for major adverse cardiovascular events, suggesting that FAI could be a promising imaging biomarker for the detection of PVAT dysfunction and coronary inflammation [41]. These findings strongly support the substantial role of PVAT in the (patho)physiology of blood vessels and CVD.

Pathogenesis of aortic aneurysm is complex and multifactorial, involving aortic remodeling-induced by genetic alterations and metabolic changes [42]. Although TAA and AAA are known to co-occur and share genetic risk factors [43], there is structural and biochemical heterogeneity between TAA and AAA with differences in aortic cellular origin and segmental growth patterns [44]. The intrinsic PVAT shows heterogeneity according to its location, resulting in likely differing paracrine PVAT factors secreted and distinct gene expression profiles [6, 15]. Several studies have indicated that inflammatory features in abdominal PVAT contribute to AAA development. For example, PVAT-derived platelet-derived growth factor-D (PDGF-D) has been shown to promote AAA formation in obese mice [14]. In humans, PVAT surrounding AAA contains necrotic adipocytes and sterile inflammatory infiltrate and robustly expresses proteases, which might result in ongoing vascular damage to the adjacent aneurysmal aortic wall [45]. Moreover, patients with infrarenal AAA have higher PVAT density around the aneurysm sac than the healthy neck of the aneurysm after adjustment for cardiovascular risk factors and other fat compartments, suggesting that PVAT contributes to AAA pathophysiology via local mechanisms [46]. However, the role of PVAT-dependent paracrine mechanism in TAA formation remains largely unexplored. We previously reported that the peri-aortic area shows high inflammation due to the absence of functional PVAT, which promotes the development of atherosclerosis [18]. In this study, we demonstrated that the absence of functional PVAT significantly exacerbates TAA formation. Additionally, the PVAT adjacent to TAA lesions in patients exhibited dysfunction and reduced PRDM16 expression. Furthermore, our findings demonstrate that the loss of PRDM16 function in PVAT leads to enhanced pro-inflammatory signaling and increased apoptosis, which are associated with the progression and severity of TAA. Supporting our findings, a recent study demonstrated that brown thoracic PVAT inhibits VSMCs apoptosis, and transplantation of thoracic PVAT significantly attenuates AAA formation compared to abdominal PVAT transplantation or sham control, likely due to the anti-apoptosis effects of the thoracic PVAT secretome [15].

PVAT regulates VSMCs functions through paracrine signaling [30]. In this study, we identified decorin as a PVAT-derived paracrine factor potentially involved in PVAT dysfunction-induced TAA development. A recent quantitative proteomic analysis demonstrated a significant increase expression of decorin in the dilated TAA zone compared with the adjacent normal aorta tissues [47], which is consistent with our findings in the PVAT and plasma of TAA patients. In addition, decorin was highly expressed in the degenerative lesions of human AAA walls, and increased in all layers of advanced AAA induced by CaCl_2_ treatment in mice [32]. Moreover, decorin induces calcification of arterial smooth muscle cell cultures and colocalizes to mineral deposition in human atherosclerotic plaque [48]. Nevertheless, both local administration of exogenous decorin and a combined intraperitoneal and intravenous injection of recombinant decorin fusion protein were shown to attenuate AAA formation in mice [31, 32], suggesting a possible dual role of decorin in AAA pathogenesis. Decorin also functions as a ligand of receptor tyrosine kinases such as EGFR and IGF-IR [49], which might also altered under pathogenic conditions and contribute to decorin’s effects on aortic aneurysmal pathogenesis. In this study, we determined that decorin was transcriptionally repressed by PRDM16, although the paracrine effects of decorin on VSMC apoptosis and their consequent impact on TAA formation remain to be fully elucidated.

It is important to note that this study’s “normal” PVAT refers to the relatively unaffected region adjacent to the aneurysmal area, as obtaining truly healthy PVAT from non-diseased individuals is not feasible. Consequently, these “normal” PVAT specimens exhibit significant inter-individual variability, as shown in Fig. 3A, where at least two patients appear to have much healthier “normal” PVAT (higher protein expression of PRDM16), while others show some degree of impairment. Notably, in cases where “normal” PVAT appears healthier, the distinction between TAA and “normal” PVAT is even more pronounced, further reinforcing our findings on PRDM16 downregulation in TAA-associated PVAT.

One limitation of this study, and probably the entire field of PVAT biology, is the lack of PVAT-specific marker genes due to its substantial similarities with BAT. The most commonly employed strategy to investigate brown-like PVAT involves generating brown adipocyte-specific knockouts using *Ucp1* promoter-driven Cre mice, which also delete genes in BAT in other locations. Thus, we could not distinguish the contributions of PVAT or BAT on the observed phenotype. However, given the unique anatomical positioning of PVAT encircling the aorta, it is reasonable to hypothesize that PVAT, rather than BAT, directly interacts with adjacent blood vessels. Future studies aiming at identifying PVAT-specific marker genes could pave the way for developing PVAT-specific knockout mouse models. Another limitation of this study is that it does not distinguish between developmental-stage-specific versus adulthood-specific roles of PPARg and PRDM16. The *Ucp1*-Cre model used in this study leads to gene ablation as soon as *Ucp1* is expressed, likely occurring during postnatal development [50]. Thus, an inducible Cre-ER^T2^ system activated by tamoxifen presents a valuable alternative methodology, which would allow temporal control over gene deletion, making it feasible to delineate the distinct roles that PPARg and PRDM16 play during different stages of PVAT development and function.

## Conclusions

In summary, through analyses of both human samples and animal models, we demonstrated that PVAT adjacent to TAA lesion regions exhibits a loss of browning characteristics. In mice, the absence of functional PVAT or its dysfunction results in increased inflammation and exacerbated TAA formation. Our findings establish a causal role for PVAT dysfunction in TAA pathogenesis and suggest that promoting PVAT browning may serve as a potential therapeutic strategy for preventing TAA progression.

## Electronic supplementary material

Below is the link to the electronic supplementary material.

Supplementary Material 1.

## Data Availability

No datasets were generated or analysed during the current study.

## Abbreviations

PVAT: Perivascular adipose tissue
TAA: Thoracic aortic aneurysm
AAA: Abdominal aortic aneurysm
WAT: White adipose tissue
BAT: Brown adipose tissue
VSMCs: Vascular smooth muscle cells
*Pparg*^BAKO^: Brown adipocyte-specific *Pparg* knockout
*Prdm*16^BAKO^: Brown adipocyte-specific *Prdm*16 knockout
PPE: Porcine pancreatic elastase
ChIP-qPCR: Chromatin immunoprecipitation-quantitative PCR
gWAT: Gonadal WAT
mWAT: Mesenteric WAT
sWAT: Subcutaneous WAT
