# Supplementary material for: Perivascular adipose tissue dysfunction contributes to thoracic aortic aneurysm development
Source: Cardiovasc Diabetol. 2025 May 21;24:223. doi: 10.1186/s12933-025-02765-x (PMC12096520; doi:10.1186/s12933-025-02765-x)
Supplement: Supplementary file 1 — Supplementary Material 1. [file 12933_2025_2765_MOESM1_ESM.docx]

**Perivascular adipose tissue dysfunction contributes to thoracic aortic aneurysm development**

Zhenguo Wang^1,†^, Wenjuan Mu^1,†^, Ruiyan Xu^1,2^, Juan Zhong^1^, Wenhao Xiong^1,2^, Xiangjie Zhao^1,3^, Xiubin Liang^1^, Yanhong Guo^1^, Jifeng Zhang^1^, Zhi-Sheng Jiang^2^, Bo Yang^4^, Y. Eugene Chen^1,4*^, and Lin Chang^1*^

^1^Department of Internal Medicine, Cardiovascular Center, University of Michigan Medical Center, Ann Arbor, MI 48109, USA

^2^Institute of Cardiovascular Disease, Key Laboratory for Arteriosclerology of Hunan Province, International Joint Laboratory for Arteriosclerotic Disease Research of Hunan Province, Hengyang Medical School, University of South China, Hengyang 421001, P. R. China

^3^Key Laboratory of Animal Cellular and Genetics, Engineering of Heilongjiang Province, College of Life Science, Northeast Agricultural University, Harbin 150030, P. R. China.

^4^Department of Cardiac Surgery, Cardiovascular Center, University of Michigan Medical Center, Ann Arbor, MI 48109, USA

^†^ Zhenguo Wang and Wenjuan Mu have contributed equally to this work.

*Correspondence: Y. Eugene Chen, [echenum@umich.edu](mailto:echenum@umich.edu); Lin Chang, lincha@umich.edu


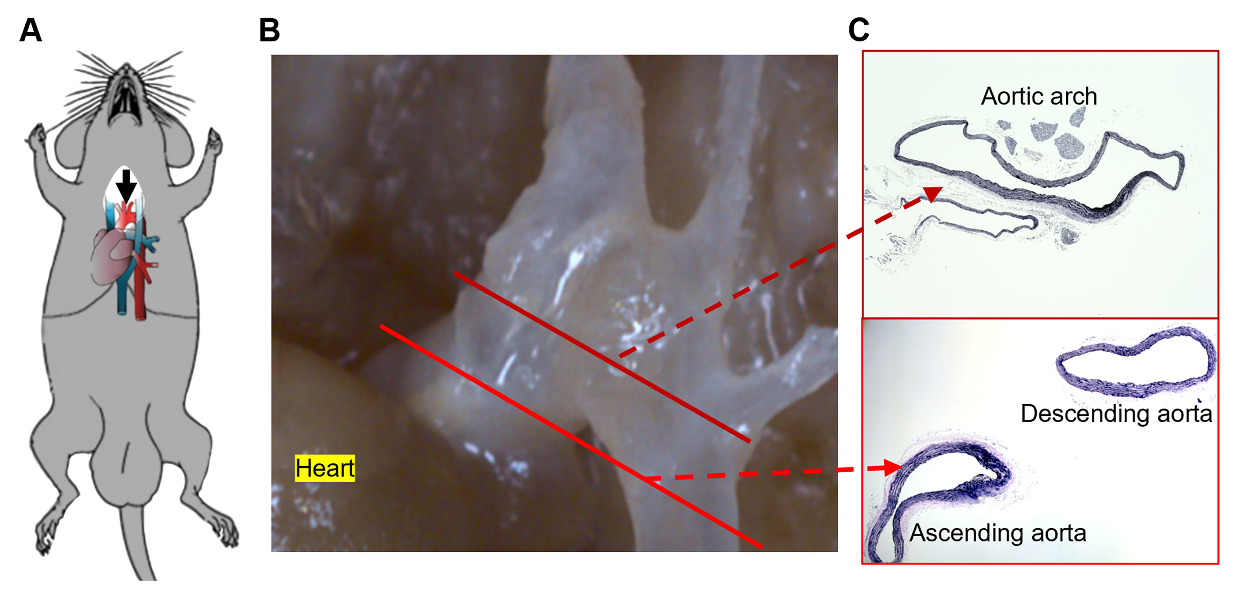


**Supplemental Fig. 1** Perivascular application of elastase-induced TAA in mice. Twelve-week-old male C57BL/6J mice were anesthetized by isoflurane inhalation. **A** The surgical site (black arrow) is shown. The aortic arch was exposed without damaging the pleura. A piece of elastase-soaked gauze (~1×1 mm) was applied was to the aortic arch for 10 minutes. The gauze was then removed, and the area was washed three times with prewarmed (37°C) 0.9% saline before wound closure. **B** The aorta was harvested two weeks post-elastase application for histological analysis. A schematic illustration depicts tissue section preparation: the longer red line represents a cross-section containing both the ascending and descending aorta, while the shorter red line indicates a cross-section of the aortic arch. **C** Verhoeff-Van Gieson (VVG) staining showing the aortic arch along with the ascending and descending aorta.


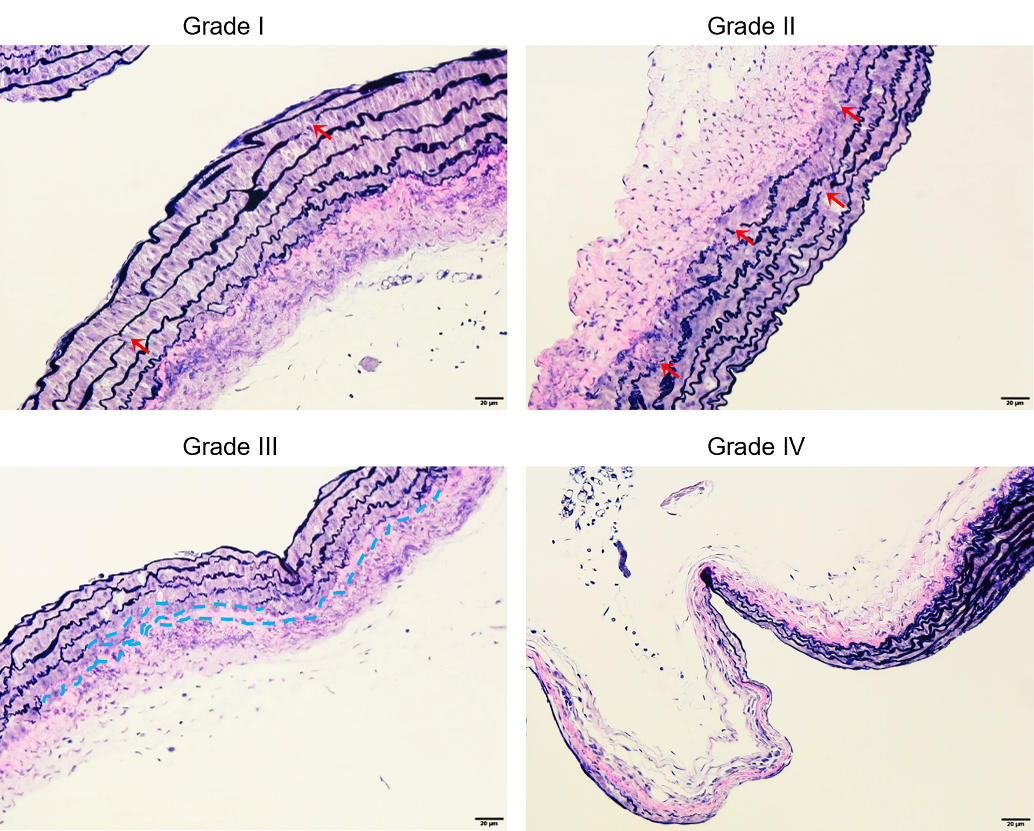


**Supplemental Fig. 2** Determine the grade of elastic fiber degradation in aortic wall in TAA model. TAA in 12-week-old mice was induced as described in the Methods section. Fourteen days later, the section of aortic wall in arch area was prepared and stained with Verhoeff-Van Gieson. The grade of elastic fiber degradation was determined as following: grade I, occasional elastic fiber broken; grade II, frequent elastic fiber broken; grade III, loss of long piece of elastic fiber; Grade IV, loss of most of elastic fibers and VSMCs. Red arrows indicate elastic fiber breaks; Blue dashed lines indicate the proposed degraded elastic fibers. Scale bars: 20 μm.


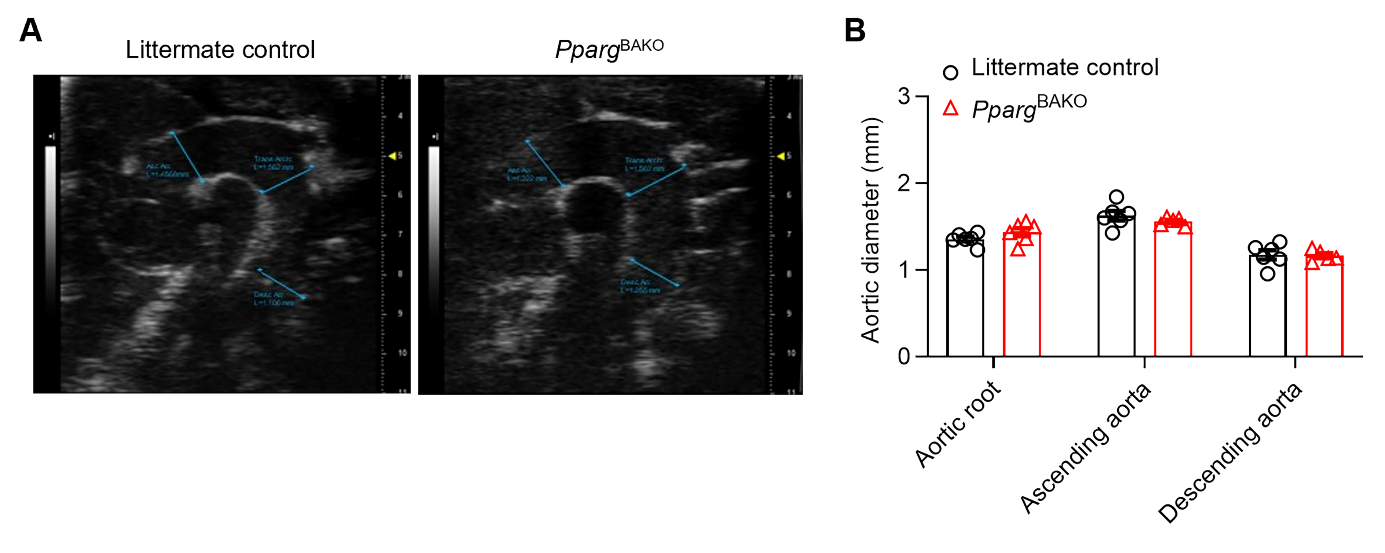


**Supplemental Fig. 3** PPARγ deficiency in brown adipocytes does not affect aortic diameter under basal conditions without elastase application. **A** Representative echocardiographic images of the ascending aorta, aortic arch, and descending aorta in *Pparg*^BAKO^ and littermate control mice. Blue lines indicate aortic diameter measurements. **B** Quantification of aortic diameter based on echocardiographic images (n = 5-6 per group). Data are presented as mean ± SEM.


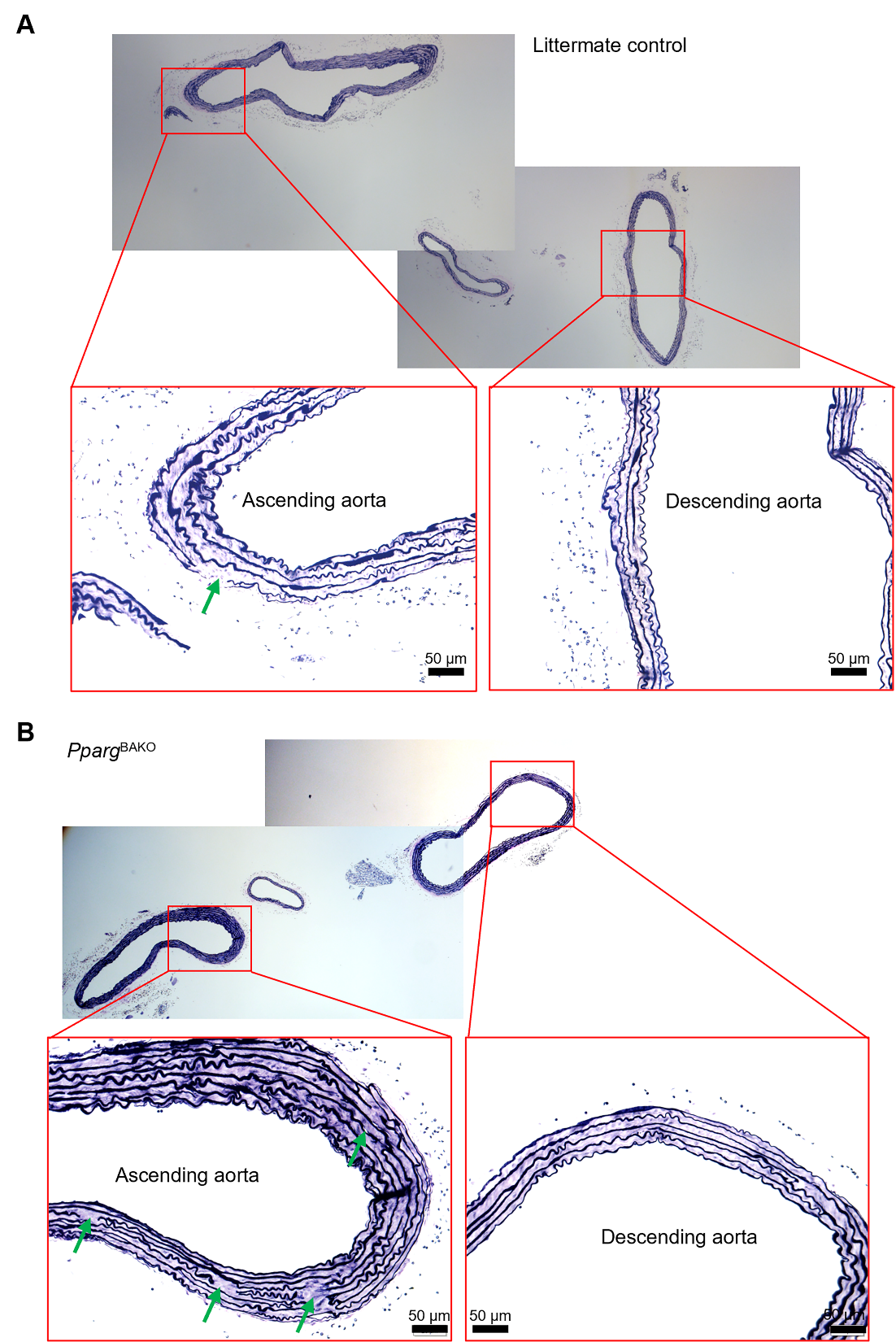


**Supplemental Fig. 4** Verhoeff-Van Gieson (VVG) staining of the ascending and descending aorta. TAA in twelve-week-old mice was induced as described in the Methods section. Fourteen days later, the section of aortic wall in the ascending and descending aorta was prepared and stained with VVG in littermate control mice (**A**) and *Pparg*^BAKO^ mice (**B**). Green arrows indicate elastic fiber breakage.


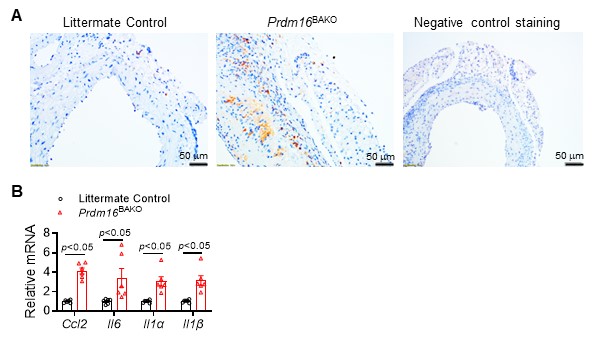


**Supplemental Fig. 5** Deficiency of *Prdm16* in brown adipocytes promoted inflammation in PVAT. **A** Staining of macrophage marker F4/80 in cross section of aortic arch together with PVAT from *Prdm16*^BAKO^ and littermate control mice. **B** The mRNA levels of inflammatory markers in the thoracic PVAT from *Prdm16*^BAKO^ and littermate control mice were measured by qRT-PCR, n=6 mice/group. **p*<0.05.

**
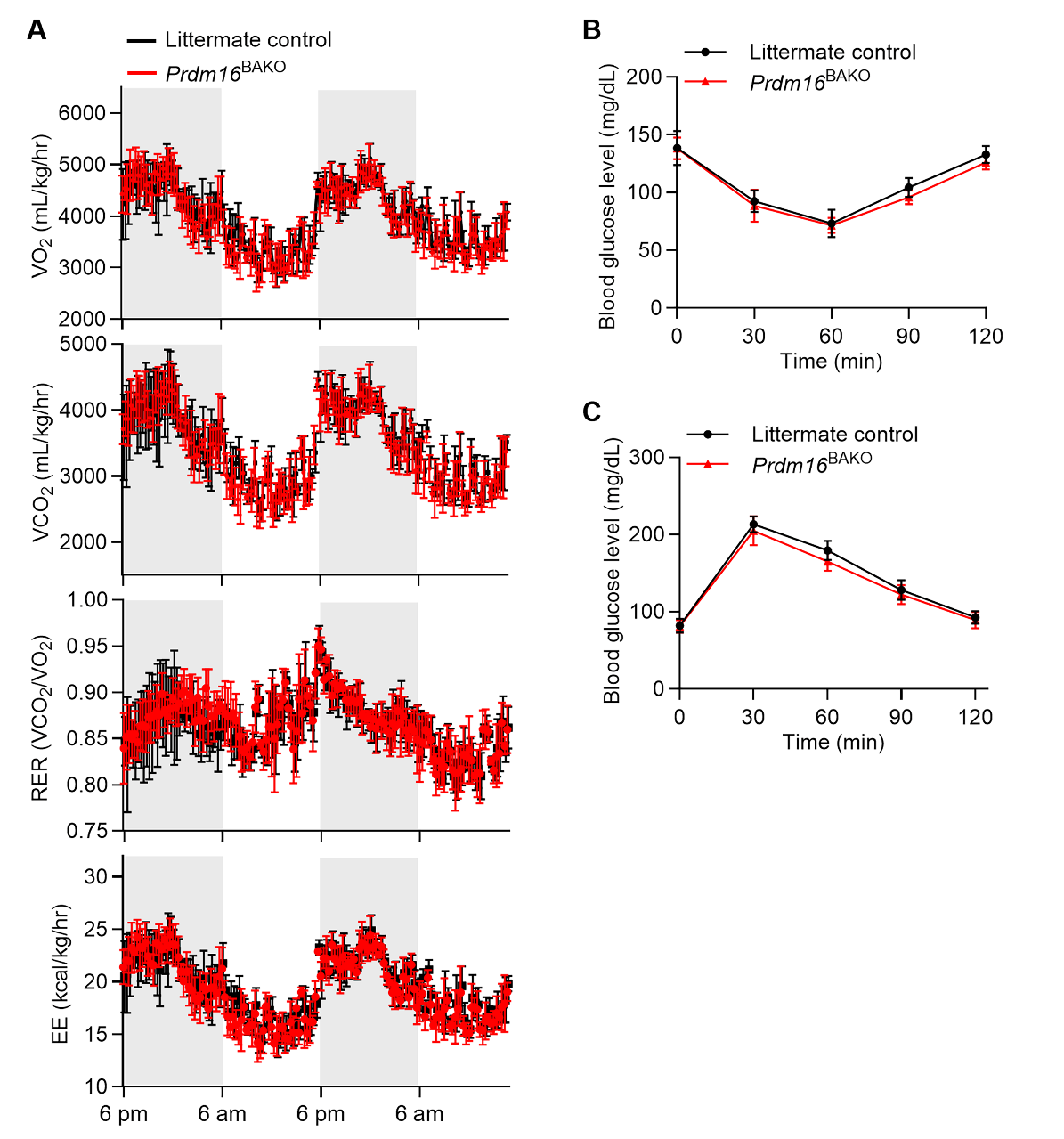
**

**Supplemental Fig. 6** Whole-body metabolism was not altered by PRDM16 deficiency in brown adipocyte. **A** Twelve-week-old male mice were acclimated to single housing for one week (22^o^C, 12 h/12 h light/dark cycles) and the O_2_ consumption rate (VO_2_) and CO_2_ production rate (VCO_2_) were recorded by the Promethion System. The respiratory exchange ratio (RER) and energy expenditure (EE) was calculated based on VO_2_ and VCO_2_. n = 5 per group. **B** Intraperitoneal insulin tolerance test (0.5 U/kg insulin). **C** Oral glucose tolerance test (2 g/kg glucose). n = 5 per group. Data are presented as mean ± SEM.


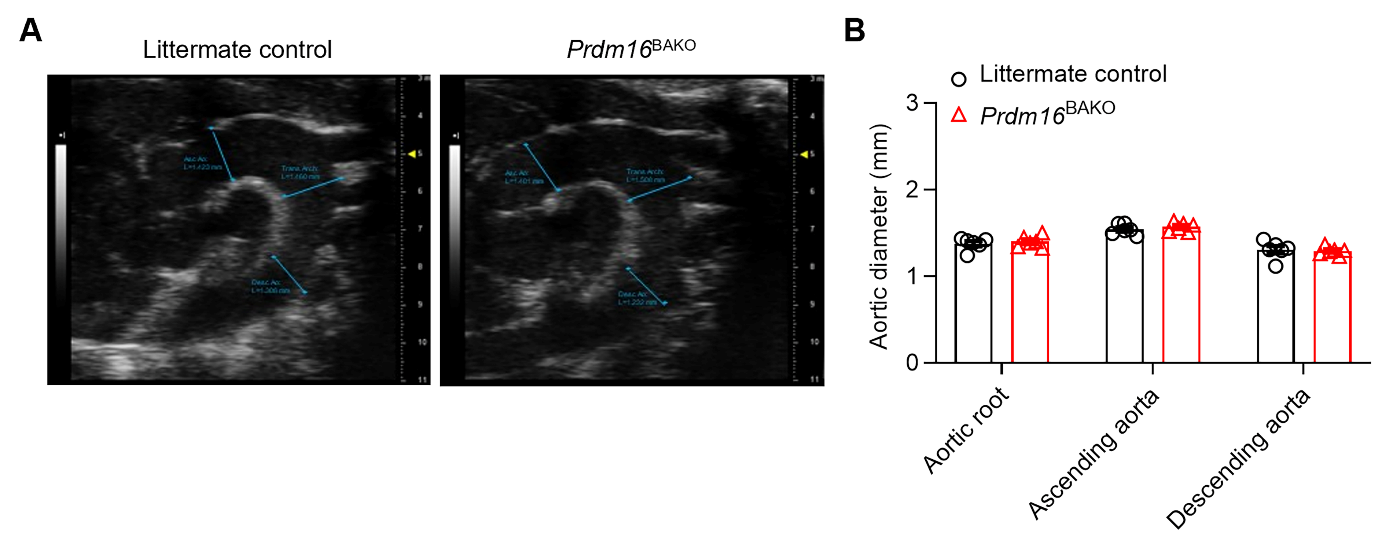


**Supplemental Fig. 7** PRDM16 deficiency in brown adipocytes does not affect aortic diameter under basal conditions without elastase application. **A** Representative echocardiographic images of the ascending aorta, aortic arch, and descending aorta in *Prdm16*^BAKO^ and littermate control mice. Blue lines indicate aortic diameter measurements. **B** Quantification of aortic diameter based on echocardiographic images (n = 6 per group). Data are presented as mean ± SEM.


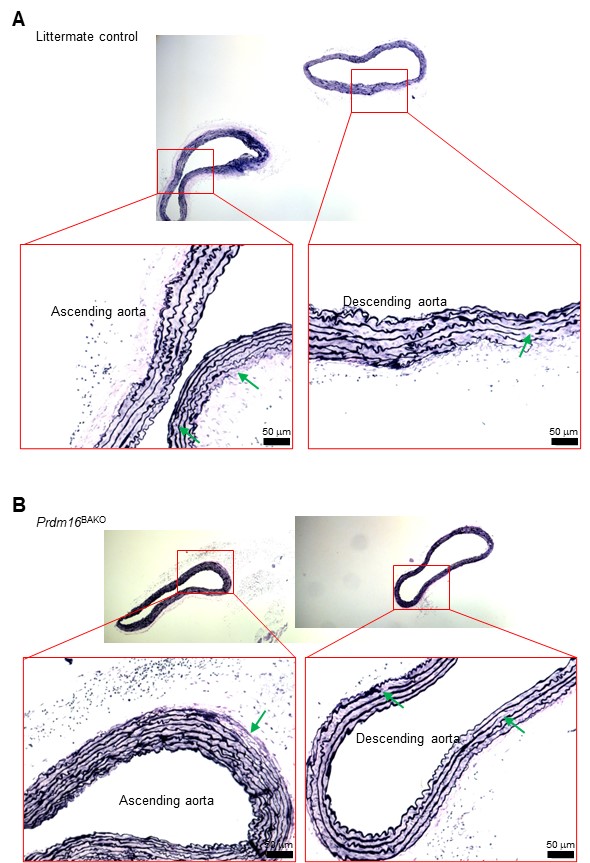


**Supplemental Fig. 8** Verhoeff-Van Gieson (VVG) staining of the ascending and descending aorta. TAA in twelve-week-old mice was induced as described in the Methods section. Fourteen days later, the section of aortic wall in the ascending and descending aorta was prepared and stained with VVG in littermate control mice (**A**) and *Prdm16*^BAKO^ mice (**B**). Green arrows indicate elastic fiber breakage.


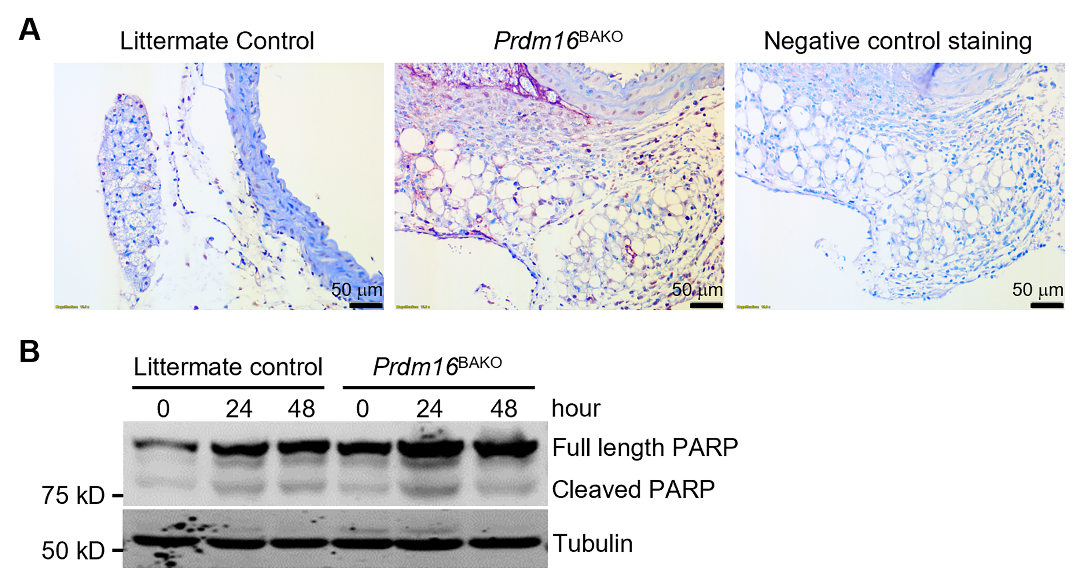


**Supplemental Fig. 9** PVAT from *Prdm16*^BAKO^ promoted VSMCs apoptosis. **A** TUNEL assay of PVAT and aorta from *Prdm16*^BAKO^ and littermate control mice. **B** Primary VSMCs isolated from C57BL/6J mice were co-cultured with PVAT from *Prdm16*^BAKO^ and littermate control mice for 24 hours. Western blot results showing increased PARP cleavage, a marker of cell apoptosis, in VSMCs co-cultured with *Prdm16*^BAKO^ PVAT for 24 or 48 hours.

Supplemental Table 1. Information of TAA patients.

| **Patient No.** | **1** | **2** | **3** | **4** | **5** | **6** |
| --- | --- | --- | --- | --- | --- | --- |
| Gender | Female | Female | Female | Male | Male | Male |
| Age (year) | 47 | 62 | 50 | 71 | 60 | 64 |
| Aneurysm size (mm) | 51 | 56 | 53 | 61 | 54 | 50 |
| Aneurysm location | Desc | Asc | Desc | Asc | Asc | Desc |
| Dissection (DeBakey) | No | No | No | No | I | III |
| Marfan syndrome | No | No | No | No | No | No |
| Bicuspid aortic valve | No | No | No | No | No | No |
| BMI (kg/m^2^) | 27 | 29 | 31 | 25 | 33 | 25 |
| SBP/DBP (mmHg) | 122/85 | 146/96 | 120/79 | 152/100 | 147/95 | 118/76 |
| History of cardiac intervention | No | No | No | No | No | No |
| Chronic renal diseases | No | No | No | No | No | No |
| Plasma total cholesterol (mg/dL) | 135 | 198 | 227 | 124 | 163 | 125 |
| Plasma LDL cholesterol (mg/dL) | 107 | 143 | 189 | 88 | 121 | 96 |
| Fasting plasma glucose (mg/dL) | 147 | 78 | 91 | 85 | 110 | 107 |
| Current smoker | No | Yes | No | No | No | No |
| Former smoker | Yes | Yes | No | No | No | No |
| β blockers | No | Yes | No | Yes | Yes | No |
| Antidiabetic drugs | Yes | No | No | No | No | Yes |

Desc: descending aorta; Asc: ascending aorta.

Supplemental Table 2. qPCR primers used in this study

| Targets | Forward primer | Reverse primer |
| --- | --- | --- |
| *UCP1* (H) | AGGTCCAAGGTGAATGCCC | TTACCACAGCGGTGATTGTTC |
| *HOXC8* (H) | CTAACAGTAGCGAAGGACAAGG | CTAGTTCCAAGGTCTGATACCG |
| *NRIP1* (H) | GGGAAGTGTTTGGATTGTGAG | AAAGTAGCTCTGATGTCATCCG |
| *PRDM16* (H) | TTCGGATGGGAGCAAATACTG | CACGGATGTACTTGAGCCAG |
| *DCN* (H) | GGACCGTTTCAACAGAGAGG | GAGTTGTGTCAGGGGGAAGA |
| *18S rRNA* (H) | GGAAGGGCACCACCAGGAGT | TGCAGCCCCGGACATCTAAG |
| *Ucp1* (M) | AAAAACAGAAGGATTGCCGAAACT | TAAGCATTGTAGGTCCCCGTGTAG |
| *Cidea* (M) | CTGTCGCCAAGGTCGGGTCAAG | CGAAAAGGGCGAGCTGGATGTAT |
| *Cox8b* (M) | TGGGGATCTCAGCCATAGTC | CTCAGGGATGTGCAACTTCA |
| *Elovl3* (M) | GGGCCTCAAGCAAACCGTGTG | GTTTGGCAGCCTTCATAGTGTAGT |
| *Dio2* (M) | ATGTAACCAGCACCGGAAAG | ATGCAGAAAGGCAGACTCGT |
| *Pgc1a* (M) | CTCCTCCCACAACTCCTCCTCATA | GGGCCGTTTAGTCTTCCTTTCCTC |
| *Otop1* (M) | GACAACCCGATGTCTGGACT | GCCAAAGACAATTTCCTCCA |
| *Tbx1* (M) | GTCAAGGCTCCGGTGAAGAAG | GCTGATTGAACTCGTCCCACA |
| *Ebf2* (M) | GGAACCGGAACGAGACCCCT | TCCCTTGGGTTTCCCGCTGT |
| *Cited1* (M) | ATGAGGAAGTGCTGATGTCG | CCTCCATCTTTAGGGACACTTG |
| *Ap2* (M) | TCACCTGGAAGACAGCTCCT | AAGCCCACTCCCACTTCTTT |
| *Adipoq* (M) | TGTTCCTCTTAATCCTGCCCA | CCAACCTGCACAAGTTCCCTT |
| *Dcn* (M) | TTGATGCACCCAGCCTGAAA | TACTTATGCTGTGCCAGCCC |
| *Dcn-ChIP* (M) | ACTTCAGAGCCCAGGAGTAATTC | TTCCCCCTTGCTTTGGAGATG |
| *Ccl2* (M) | GGTCTTCAGCACCTTTGAATG | ATTAAGGCATCACAGTCCGAG |
| *Il6* (M) | TAGTCCTTCCTACCCCAATTTCC | TTGGTCCTTAGCCACTCCTTC |
| *Il1a* (M) | CGAAGACTACAGTTCTGCCATT | GACGTTTCAGAGGTTCTCAGAG |
| *Il1b* (M) | TCCTGTGTAATGAAAGACGGC | ACTCCACTTTGCTCTTGACTTC |

H, human; M, mouse. Primers were synthesized at Integrated DNA Technologies (IDT).
